# Levosimendan in the Treatment of Patients with Severe Septic Cardiomyopathy

**DOI:** 10.3390/life13061346

**Published:** 2023-06-08

**Authors:** Vasiliki Tsolaki, George E. Zakynthinos, John Papanikolaou, Vasileios Vazgiourakis, Kyriaki Parisi, George Fotakopoulos, Demosthenes Makris, Epaminondas Zakynthinos

**Affiliations:** 1 Intensive Care Unit, University Hospital of Larissa, University of Thessaly Faculty of Medicine, 44110 Larissa, Greece; vasvazg@yahoo.com (V.V.); kyriakiparisi@yahoo.com (K.P.); dimomakris@uth.gr (D.M.); ezakynth@yahoo.com (E.Z.); 2 Third Cardiology Clinic, University of Athens, Sotiria Hospital, 11527 Athens, Greece; gzakynthinos2@gmail.com; 3 Cardiology Department, Trikala Hospital, 42131 Trikala, Greece; y_papanikolaou@hotmail.com; 4 Neurosurgical Department, University Hospital of Larissa, 44110 Larissa, Greece; gfotakop@yahoo.gr

**Keywords:** circulatory shock, levosimendan, septic cardiomyopathy, survival, treatment

## Abstract

(1) Background: The optimal treatment of septic cardiomyopathy (SCM) remains questionable. The aim of the study was to compare the treatment of SCM based on levosimendan versus the best available therapy. (2) Methods: We conducted an observational study including patients with severe septic cardiomyopathy and circulatory failure. (3) Results: Fourteen patients (61%) received levosimendan, and nine received other treatments. The patients in the levosimendan group were more severely ill [APACHE II: 23.5 (14, 37) vs. 14 (13, 28), respectively, *p* = 0.012], and there was a trend for more decompensated LV function depicted by the LVEF [15% (10, 20) vs. 25% (5, 30), respectively, *p* = 0.061]. However, they presented a significantly higher increase in LVEF after seven days [15% (10, 20) to 50% (30, 68) (*p* < 0.0001) vs. 25% (5, 30) to 25% (15, 50) (*p* = 0.309), and a significantly higher decrease in lactate levels during the first 24 h [4.5 (2.5, 14.4) to 2.85 (1.2, 15), *p* = 0.036 vs. 2.9 (2, 18.9) to 2.8 (1, 15), *p* = 0.536]. Seven-day survival (64.3% vs. 33.3%, *p* = 0.424) and ICU survival (50% vs. 22.2%, *p* = 0.172) were higher in the first group, although differences did not reach statistical significance. The degree of left ventricular impairment and the magnitude of EF improvement by the seventh-day post-SCM onset were associated with mortality in regression analysis. (4) Conclusions: Our study presents main hemodynamic data supporting the possible efficacy of levosimendan treatment in patients with severe SCM.

## 1. Introduction

Sepsis is a life-threatening condition of the dysregulated host response to an infection that may lead to multi-organ dysfunction [1]. Septic shock is associated with a mortality of up to 50% [2]. Among the organs being affected, the heart is centrally involved. Characteristic features of septic cardiomyopathy (SCM) include one or more of the following: (1) left ventricular dilatation with normal or low-filling pressure, (2) reduced ventricular contractility, and (3) right ventricular dysfunction or left ventricular (systolic or diastolic) dysfunction with a reduced response to volume infusion [3,4,5]. However, the exact prevalence of septic SCM is unknown, and the reported incidence varies between 10 and 70% due to the lack of clear SCM definition criteria, pre-existing cardiac function, and criteria to promptly direct the investigation for its presence during a septic episode [3]. The diagnosis of a failing heart is further complicated by the significant and dynamic alterations in systemic hemodynamics during sepsis (with variable preload and afterload conditions) [6]. Thus, not only the incidence but also the actual time septic cardiomyopathy ensues during the course of sepsis, and the period needed for the full establishment of cardiogenic shock is unknown.

The effect on mortality of sepsis-induced myocardial dysfunction has long been debatable, and there are data supporting no mortality increase. SCM is mainly a transient myocardial impairment lasting 7–10 days during the course of severe sepsis and septic shock [7,8]. On the other hand, the “afterload-related myocardial performance”, indicating the specific myocardial contractility, adjusted for the present degree of systemic vascular resistances has been evaluated as a measure to unravel SC presence in an apparently normal functioning heart [6,9]; the degree of afterload-related myocardial performance impairment has been linked to survival, even in patients with sepsis apart from septic shock [9]. Furthermore, in patients with severe myocardial dysfunction and decreased cardiac index during sepsis, the mortality might exceed 80% [10,11]. Regarding the management of SCM, there are no evidence-based recommendations. The most commonly suggested approach is to treat the underlying disease process, which will lead to the improvement in cardiac function, accompanying sepsis improvement [3]. Yet, ongoing research improving our understanding of the underlying pathophysiological mechanisms implicated in SCM has provided the rationale for certain treatment options. Levosimendan is a calcium sensitizer that functions not only as an inotrope (increasing cardiac output without increasing myocardial oxygen demands) but as a lusitrope, presenting anti-inflammatory, anti-oxidant, and anti-apoptotic effects. A randomized controlled trial (RCT), the Levosimendan for the Prevention of Acute Organ Dysfunction in Sepsis (LeoPARDS study), failed to demonstrate any benefit from the addition of levosimendan in the treatment of patients with sepsis in terms of organ dysfunction improvement [12]. A subgroup analysis of the Leo-PARDS trial, including patients with septic shock and increased cardiac biomarkers (troponin, NT-proBNP), confirmed the lack of treatment efficacy with the addition of levosimendan [13]. Yet, patients with sepsis or septic shock present a variable cardiac function ranging from hyper to normo and hypokinesia; thus, the addition of an inotrope in patients with left ventricular hyperkinesia or normokinesia might be of no benefit [14]. Moreover, cardiac biomarkers may increase during sepsis, irrespective of cardiac dysfunction [15]. Consequently, the negative results from the large RCT might be attributed to the inclusion of a mixed population concerning the degree of myocardial impairment [12]. The aim of the present study is to evaluate the effectiveness of the addition of levosimendan in the treatment of septic shock patients with severe SCM, confirmed with echocardiography [left ventricular ejection fraction (LVEF) < 30%].

## 2. Materials and Methods

In this prospective observational study, we included mechanically ventilated patients with septic shock and severely impaired myocardial function admitted to the Intensive Care Unit of the University Hospital of Larissa between November 2019 and March 2023. The study was approved by the local ethics committee (55949/2020). The inclusion criteria were as follows (1). Age > 18 years, (2). Signs of Sepsis presence according to Sepsis III definition [2], (3). Circulatory shock [need for vasopressors to maintain Mean Arterial Pressure (MAP) > 65 mmHg and lactate levels > 2 mmol/lt] after initial resuscitation with fluids (4). LVEF < 30% after fluid resuscitation. Patients were entered if they presented with severe SCM during the course of a septic episode leading to ICU admission or if presented during the ICU stay. The exclusion criteria were: (1). Patients with a pre-existing history of severe heart disease (valvular heart disease, dilated cardiomyopathy, coronary heart disease, myocardial infarction, or known heart failure). (2). Obstructive shock (tamponade, massive pulmonary embolism, or tension pneumothorax). Patients were included irrespective of the treatment they received, the decision for which was at the discretion of the treating physician. Patients receiving levosimendan were compared with patients that had received any other kind of treatment. For patients under levosimendan, there was no loading dose preceding the 24 h administration of the drug. All the patients had subclavian or jugular central venous pressure [for central venous oxygen saturation (ScvO_2_) measurements and evaluation of the difference in arteriovenous CO_2_ pressure (Pa-vCO_2_)]. All the patients received vasopressors to maintain a MAP between 65–70 mmHg.

Values that were recorded are:

Demographics: age, sex, source of infection (intraabdominal, urinary tract, lung infection) (including if the infection was community or healthcare-associated), or bacteremia with an undetermined origin, illness severity scores (SOFA, APACHE II).

Hemodynamics: mean arterial pressure (MAP), heart rate, heart rhythm, lactate, ScvO_2_, Pa-vCO_2_, Central Venous Pressure (CVP), and LVEF. The measurements indicate the values obtained during the worst cardiac function assessment (evaluated by left ventricular ejection fraction). The indication for echocardiography was hemodynamic deterioration (need for vasopressor dose or lactate levels) despite stabilization after the initial resuscitation with fluids or blood products, where indicated. Baseline LVEF (before SCM onset) is reported. Also, the time that elapsed from the last known (baseline) cardiac function until the documentation of severe SCM is mentioned.

SCM trajectory: Lactate levels, ScvO_2_, and noradrenaline dose measured at different time intervals indicated as H0 (initial measurements upon SCM), after 12 h (H12), 24 h (H24), and 72 h (H72). On the seventh day after SCM onset, LVEF was evaluated and presented. Moreover, the changes (initial value-final value)/initial value × 100% are presented for each time point of the serial measurements.

Outcome: SCM resolution, survival upon the seventh ICU day (D7 survival), and ICU survival (survival until the patient was discharged from the ICU).

Definitions:

Severe SCM: LVEF < 30% in the course of a septic episode with lactate levels > 2 mmol/lt.

Severe SCM onset: the time the patients presented with LVEF < 30% (which was acute in onset, as the baseline LVEF upon ICU admission had been obtained).

SCM was considered to have improved or resolved (SCM improvement/resolution) if: 1. the patients presented LVEF > 45% despite the need for vasoactive drugs; 2. The patient was weaned from vasopressors with EF improvement, even if it did not reach the above value of 45% (the improvements in EF are significant considering the increase in systemic vascular resistances and their effect on left ventricular contractility [6]); or 3. Improved EF compared to the EF at the SCM onset, but <45% if the value remained stable in at least three evaluations during a period of 6 days after improvement.

**Statistical analysis**

Data were tested for normality with the Kolmogorov–Smirnov test. Non-normally distributed variables were expressed as medians (minimum, maximum values). Comparisons between the levosimendan group and the other treatment group were completed using the Mann–Whitney U test. Multiple group comparisons between serial measurements of noradrenaline levels, ScvO_2,_ and lactate levels were performed with a one-way analysis of variance (ANOVA). ICU survival was estimated by the Kaplan–Meier test for patients receiving levosimendan or other treatment and compared with a log-rank test. Cox regression analysis was performed in order to evaluate the effect of different factors on survival in the two treatment groups. Statistical analyses were performed using SPSS version 26.0 (IBM), considering *p* < 0.05 to be statistically significant.

## 3. Results

During the study period, 1050 patients were admitted to the ICU. Of these patients, 778 presented with at least one septic episode during the course of their ICU stay. From these, 23 patients with septic shock presenting with severe SCM were included in the study. The incidence of severe SCM in the cohort of septic patients included in the study is 3%. The median age was 61 (30, 84) years, and 14 (61%) were male (Table 1).

In 11 (48%) of the patients, the infection leading to SCM was community-acquired, and the most common sources were the abdomen (35%) and the lung (22%). In 19/23 (82.6%) of the patients, baseline left ventricular function was known as it had been evaluated upon ICU admission. In the remaining four patients, severely impaired LVEF < 30% was present upon ICU admission (the reason for admission was septic shock in all). None of the patients had any known history of heart failure. Two received levosimendan (initial EF 20% and 15%), and the other two (initial EF 10% and 30%) were managed with noradrenaline. By the seventh day in ICU, the LVEF value was 35%, 68%, could not be evaluated in the third patient as he passed the third day, and 45%, respectively.

The median baseline LVEF was 50% (40, 58). The median time for SCM establishment (from the last known cardiac function level) was 7.5 (2, 26) hours. All patients presented sepsis-induced cardiogenic shock as depicted by a depressed EF [median EF 15% (5, 30)], ScvO_2_ [median 62% (38, 74)], and increased Pa-vCO_2_ [median 10.8 (1, 15) mmHg] and lactate level [median 4.4 (2, 18.9) mmol/lt]. Of the 23 patients, 11 (47.8%) presented atrial fibrillation on SCM onset before any inotropic therapy (levosimendan or dobutamine was initiated), except in one patient AF started after levosimendan infusion onset. Ten patients (43.5%) presented non-specific T wave changes. The median troponin levels were 0.38 (0.01, 10) ng/mL. In four patients, coronary angiography was performed, revealing no critical coronary stenosis to warrant an intervention.

All the patients received noradrenaline and vasopressin to maintain MAP between 65 and 70 mmHg. Levosimendan initiation was at the discretion of the treating physician. Fourteen patients (61%) were treated with levosimendan, and the rest received dobutamine (n = 2) or only noradrenaline (Table 2). Four patients in the levosimendan group received dobutamine additionally.

Patients in the levosimendan group were more severely ill compared to the other treatment group [APACHE II: 23.5 (14, 37) vs. 14 (13, 28 8, 33), respectively, *p* = 0.012] and there was a trend for more decompensated LV function depicted by the LVEF [15% (10, 20) vs. 25% (5, 30), respectively, *p* = 0.061] and the Pa-vCO_2_ [ 11 (1, 15) vs. 9 (3, 14), *p* = 0.069], one of the indices indicating more severe circulatory failure [15]. Concerning the response to the treatment administered, patients in the levosimendan group presented a significantly higher increase in LVEF after seven days [15% (10, 20) to 50% (30, 68) (*p* < 0.0001) vs. 25% (5, 30) to 25% (15, 50) (*p* = 0.309) *p* = 0.02 (for the EF change comparison between the two groups], and a significantly higher decrease in lactate levels during the first 24 h [4.5 (2.5, 14.4) to 2.85 (1.2, 15), *p* = 0.036 vs. 2.9 (2, 18.9) to 2.8 (1.2, 12.9), *p* = 0.536]. Concerning the ScvO_2_ values, there was a significant increase in the levosimendan group across the first 72 h [*p* = 0.002 (ANOVA)] and by the seventh day (*p* = 0.001), while in the other treatment group the change did not reach statistical significance (*p* = 0.056), although the values upon SCM onset did not differ.

**Figure 1:**
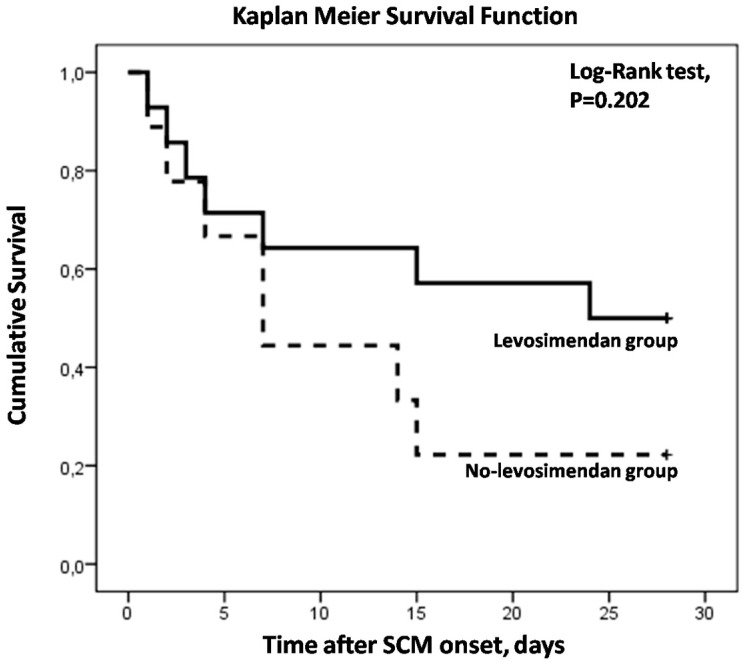
Kaplan–Meier Survival curves showing the relation between 28-day survival and treatment with levosimendan or not in septic cardiomyopathy.

SCM was resolved in 9 patients (64.3%) in the levosimendan group vs. 2 (22.2%) in the other treatment group (*p* = 0.054) (Table 3). Seven-day survival (64.3% vs. 33.3%, *p* = 0.424) and ICU survival [50% vs. 22.2%, *p* = 0.202 (Log Rank test)] were higher in the first group, although differences did not reach statistical significance (Table 2, Figure 1). Considering the patients receiving combination treatment with levosimendan and dobutamine, they all had a favorable outcome concerning SCM improvement and 3/4 (75%) were discharged from the ICU. Of the patients receiving only dobutamine, one had a favorable outcome and was discharged from the ICU, while the second died on the fourth-day post-SCM-onset.

In one patient, in the levosimendan group, an intra-aortic balloon pump was inserted. This patient had refractory shock despite the interventions (lactate > 15 mmol/lt) and ultimately died on the 10th ICU day. In Cox regression analysis, the clinical factors affecting survival were the ejection fraction that the patients presented during the episode of septic cardiomyopathy [HR 0.887 (95% CI 0.790 to 0.996), *p* = 0.042] and the degree of EF improvement by the seventh day. On the contrary, the severity scores were not identified as predictors (Table 4).

## 4. Discussion

In the present study, we found that in patients presenting with severe septic cardiomyopathy, survival was affected by the degree of left ventricular systolic function impairment and the degree of subsequent improvement: the lower the EF and the lower the improvement in EF by the seventh post-SCM day, the worse was the survival function. Moreover, we found that in severe SCM complicating the course of a septic shock episode, the treatment with levosimendan was probably an efficacious treatment in improving circulatory failure, as depicted by the faster lactate clearance and the normalization of the ScvO_2_. The improvement in left ventricular ejection fraction was higher. More patients presented a favorable outcome concerning the resolution of SCM, while patients in the levosimendan group presented higher survival rates, although the difference did not reach statistical significance. It should be mentioned, though, that patients in the levosimendan group were sicker, presenting higher APACHE II scores upon ICU admission and worse LVEF during the septic episode, issues that might further strengthen the value of levosimendan in SCM.

Reversible myocardial dysfunction is frequently noted in the course of a septic episode, involving 10–70% of patients. While SCM is common, cardiogenic shock due to sepsis has not been well clarified, especially in terms of prevalence and outcomes [3]. In our study, including patients with severe SCM, meaning patients with circulatory failure (EF < 30%, and Lac > 2 mmol/lt), the deterioration in heart function leading to severe SCM occurred within 7.5 h from the last known echocardiographic study (usually upon ICU admission). An initial myocardial function might also indicate a degree of SCM, considering the reduced afterload in this subset of septic patients [6,9]. Patients receiving levosimendan presented a significant resolution in the signs of circulatory failure. The lactate levels presented a faster clearance, while ScvO_2_ normalized significantly only in the levosimendan group. ScvO_2_ can depict the adequacy of cardiac index and tissue perfusion at the bedside and has been shown to improve in patients with acute heart failure receiving levosimendan compared to dobutamine [16,17]. However, this is the first study to depict the levosimendan benefits in global oxygenation in septic shock patients with SCM through measurements of the ScvO_2_. In relation to this, Morelli et al. showed that gastric mucosal perfusion increased in 15 patients with SCM after 24-h levosimendan administration [18]. The noradrenaline dose did not differ between the two groups, nor did the patients in the levosimendan group warrant a higher dose of vasopressors during the following days. The worsening in the vasodilatory shock is a feared side effect of levosimendan, preventing the ease in the choice of this drug in septic patients with already established vasodilatory shock. Moreover, the patients treated with levosimendan presented more significant improvements in LVEF on the seventh day after SCM onset, although the EF during the seventh day did not differ between the two groups. When analyzing these results, the significant interplay between the left ventricular contractile function upon a variable afterload level should always be considered [6].

Studies on SCM have reported conflicting results concerning survival, while the impact of the hyperkinetic myocardium has been emphasized on adverse outcomes [7,14,19]. It should be highlighted, though, that the definition of SCM is significantly variable and encompasses a diverse population of depressed left ventricular function, including patients presenting LVEF < 50% or a 10% decrease in LVEF compared to a known baseline value [5]. Other studies have reported a decreased survival rate in patients with more severe impairments in left ventricular performance. Poelaert et al. reported a survival rate of only 17% in patients with LVEF below 40% [11]. Recently, Brechot et al. described the outcomes of 212 patients with cardiogenic shock due to septic cardiomyopathy, 82 of whom were treated with Extra Corporeal Membrane Oxygenation (ECMO). All the included patients presented severely reduced LVEF (<35%), severe hyperlactatemia (>4 mmol/lt), and had an inotrope score of at least 75 μg/kg/min. The survival rate in the cohort not receiving ECMO was only 25% which is in accordance with the survival rate seen in the cohort of patients in the present study not receiving levosimendan. There are no data comparing one treatment modality to another, or even combinations of treatment modalities, but it seems logical that the availability of treatments will drive the choice. In our cohort, three out of the four patients receiving a combination of inotropes (levosimendan and dobutamine) had a favorable outcome and were discharged from the ICU. Certainly, this is only a small subset of patients. Thus, we can not conclude on the efficacy of combination treatments. Yet, we found that the degree of EF improvement is a key variable affecting survival. Thus, the research should focus on treatments enhancing myocardial function for better outcomes. Whether this is translated in the use of a combination of inotropic treatments shall be evaluated in future trials.

Septic cardiomyopathy is a sepsis-associated syndrome unrelated to ischemia. Many mechanisms have been proposed to result in cardiac dysfunction during a septic episode. Mitochondrial dysfunction, excessive release of inflammatory cytokines, oxidative stress, free-radical production, altered nitric oxide metabolism, endogenous damage-associated molecular patterns, pathogen-associated molecular patterns, and components of the complement cascade have all been proposed [20,21,22]. However, there is no consensus on the pathophysiology, even though calcium desensitization seems to present one of the most important mechanisms [23,24]. When the myocardial dysfunction warrants inotropic therapy, the clinician should choose between dobutamine, epinephrine and levosimendan. Catecholamines have shown limited efficacy, while they are accompanied by an increased risk of arrhythmias and myocardial oxygen demand [25]. Hernandez et al., in a randomized controlled trial evaluating the microcirculatory perfusion in septic shock patients treated with dobutamine, reported negative results, despite an increase in the cardiac index in the dobutamine-treated patients [26]. Furthermore, there is no study reporting a mortality benefit with inotropic therapy [27].

Levosimendan seems an attractive treatment modality for severe SCM. As a unique noncatecholamine inodilator, it improves cardiac contractility by increasing the sensitivity of troponin C to calcium, limiting the risk of arrhythmogenesis or excessive oxygen demand [28]. Contractility augmentation is not associated with increases in calcium transients or intracellular calcium. Its action is independent of beta receptor activation and, thus, is not affected by pretreatment with beta-blocker agents [29]. Moreover, levosimendan has been associated with cardioprotection and mitigation of ischemia/reperfusion injury through its action in K_ATP_ channels on the mitochondrial inner membrane [30,31]. In addition to calcium sensitization, levosimendan acts as an inodilator, mediating the opening of ATP-dependent potassium channels in vascular smooth muscle cells in different vascular beds [32]. This may be associated with hypotension but also with an increase in specific organ perfusion [33]. Additional actions include anti-inflammatory effects, reduction of inflammatory cytokines and oxidative stress levels, and anti-apoptotic actions. Until today, there is a lack of robust evidence on the efficacy of levosimendan in septic patients with SCM, mainly arising from much confusion on the interpretation of the results of many studies. This is depicted in the Surviving Sepsis Campaign guidance against the use of levosimendan in septic shock patients with cardiac dysfunction [34]. The recommendation is mainly based on the results of a large randomized controlled trial, the LeoPARDS study, evaluating the efficacy of levosimendan administration in the prevention of acute organ dysfunction in patients with septic shock receiving vasopressors for at least four hours. The trial failed to present a benefit in the treatment group, while the patients were less likely to be weaned from the ventilator and had a higher risk for supraventricular arrhythmias [12]. However, the patients enrolled were not evaluated for cardiac dysfunction. Thus, the results cannot address the effect of levosimendan on SCM and mainly in severe SCM, as in our patient group with markedly reduced left ventricular ejection fraction. Moreover, the authors realized this drawback and conducted a subsequent analysis of the same study focusing on patients with cardiac dysfunction. Yet, the myocardiopathy was documented through biomarker elevation and not objective criteria for myocardial performance evaluation [13]. The results should be interpreted with great skepticism as they certainly do not answer the question of levosimendan’s benefit for patients with SCM.

Two meta-analyses, including almost the same randomized controlled trials, reported conflicting results concerning patient outcomes. Zangrillo et al. reported a significant reduction in mortality in patients with severe sepsis and septic shock receiving levosimendan, while Bhattacharjee et al. failed to observe evidence of advantage on mortality at longest follow-up and the length of ICU stay [35,36]. Both meta-analyses included studies concerning patients with sepsis and septic shock and did not focus on patients with septic cardiomyopathy. Only 3/6 studies reported that they included patients with EF below a variable level of 65%, 45%, and 35%. Thus, the population included is rather heterogenous, and no clear conclusion can be drawn for the addition of levosimendan in the treatment armamentarium of SCM. In our study, including a more homogenous population of septic patients with severely reduced left ventricular systolic function, there was a trend for increased survival in patients treated with levosimendan, yet the conclusion is not straightforward due to the small sample size and the confounding effects of sepsis and septic shock on the final outcomes.

In the present study, we showed that the degree of EF impairment and the amplitude of EF’s improvement are significant factors affecting mortality. Interestingly, although patients in the levosimendan group presented a trend for lower EF upon SCM onset, levosimendan treatment led to a higher survival benefit compared to patients receiving other treatments. Thus, levosimendan treatment might significantly affect survival even in patients with the worst EF. Recently, Sun et al., in a randomized controlled study of thirty patients with severe SCM, found that levosimendan led to significant increases in the myocardial performance of the treated patients compared to those receiving dobutamine, presented a decreased need for noradrenaline dose after 24 h of treatment, while additionally, there was a normalization in the levels of cardiac biomarkers [37]. The patients included presented a mean EF of 28%, higher than the EF of patients in our cohort. This difference might explain the mortality difference between the two groups not receiving levosimendan (28-day mortality in the Sun et al. study was 53% vs. 77.8% ICU mortality in our study) and further emphasizes the advantage of the treatment in patients with severely reduced EF.

Certain limitations should be addressed. First, this is not a randomized trial. Therefore, the results should be interpreted with caution. Non-randomization imbalances were evidenced in the greater illness severity of patients in the levosimendan group (APACHE II score, initial EF upon SCM onset). Yet, the differences presented between groups further strengthen the benefit of levosimendan treatment, as it led to more significant improvement in LVEF, and the resolution of cardiogenic shock, as depicted by the faster lactate clearance and the increase in ScvO_2_ values. The small sample size and the monocentric character of the study are other limitations, but our study included a homogenous group of septic shock patients with severely depressed left ventricular function. Other treatment modalities, such as ECMO or an intra-aortic balloon pump for the management of severe SCM, have not been evaluated, as they are not widely available, and they require significant expertise.

## 5. Conclusions

Our study presents the main hemodynamic data supporting the possible efficacy of levosimendan treatment in patients with severe SCM. Levosimendan might reverse the adverse signs of circulatory failure in patients with circulatory failure due to cardiogenic shock in the course of a septic episode, while it could result in a higher likelihood of improvement in the left ventricular function. The degree of left ventricular impairment seems to be associated with the mortality rate in patients with SCM.

## Figures and Tables

**Table 1:** Baseline characteristics of the whole cohort upon SCM ensue.

	Whole Group (n = 23)	Levosimendan Group (n = 14)	Other Treatment Group (n = 9)	*p*-Value
Age	61 (30, 84)	60 (30, 79)	76 (52, 48)	0.360
Sex (male)	14 (61%)	9/14 (64.3%)	5/9 (55.6%)	0.675
SOFA score	10 (5, 24)	10 (7, 24)	10 (5, 15)	0.440
APACHE II	19 (8, 37)	23.5 (14, 37)	14 (8, 33)	0.012
Community-acquired infection [n (%)]	11 (48%)	7 (50%)	4 (44%)	0.795
Source of infection †: IAI [n (%)]	8 (35%)	6 (43%)	2 (22%)	0.072
Source of infection †: lung [n (%)]	5 (22%)	4 (29%)	1 (11%)	
Source of infection †: UTI [n (%)]	2 (9%)	1 (7%)	1 (11%)	
Blood Stream Infection (BSI) with no obvious source	8 (35%)	3 (21%)	5 (56%)	
Hours to severe SCM ^	7.5 (2, 26)	6 (3, 26)	14 (2, 18)	0.160
BPM	120 (54, 155)	126 (100, 155)	120 (54, 150)	0.411
Baseline EF (%)	50 (40, 58)	54 (45, 56)	50 (40, 58)	0.721
EF upon SCM episode (%)	15 (5, 30)	15 (10, 20)	25 (5, 30)	0.061
Noradrenaline H0 (μg/kg/min)	0.87 (0.35, 1.67)	0.91 (0.49, 1.63)	0.75 (0.35, 1.67)	0.356
ScvO_2_ (%)	62 (38, 74)	58 (38, 74)	65 (50.6, 68)	0.424
Pa-vCO_2_ (%)	10.8 (1, 15)	11 (1, 15)	9 (3, 14)	0.069
CVP (mmHg)	14.5 (1, 26)	15 (1, 26)	14 (3, 18)	0.578
TNI * (ng/mL)	0.38 (0.01, 10)	0.14 (0.01, 10)	0.56 (0.01, 10)	0.867
Lactate H0 (mmol/lt)	4.4 (2, 18.9)	4.5 (2.5, 14.4)	2.9 (1, 18.9)	0.412

APACHE II; Acute Physiologic Assessment and Chronic Health Evaluation, BPM; Beats Per Minute, CVP; Central Venous Pressure, EF; Ejection Fraction, IAI; Intra-Abdominal Infection, Pa-vCO_2_; arteriovenous difference of partial dioxide pressure, SCM; Septic Cardiomyopathy, ScvO_2_; Superior Vena Cava Oxygen Saturation, SOFA; Sequential Organ Failure Assessment, TNI; Troponin, UTI; Urinary Tract Infection, † Either community-acquired or hospital-acquired. ^ The hours to severe SCM establishment are measured from the time of the last known heart function (usually ICU admission) until the echocardiographic diagnosis of severe SCM. * Troponin normal values < 0.02 ng/dL.

**Table 2:** Course of circulatory failure in the two groups of patients during the septic episode.

		T_scm_	T_12h_	T_24h_	T_72h_	T_D7_	*p*-Value
Heart Rate	Levosimendan Group	133 (65, 155)	124 (65, 159)	124 (67, 160)	109 (75, 124)	83 (54, 128)	0.005
	“Other treatment” Group	120 (54, 150)	112 (56, 167)	113 (78, 129)	100 (67, 136)	96.5 (55, 119)	0.288
MAP	Levosimendan Group	65 (60, 68)	65 (61,67)	66 (60, 68)	67 (62, 69)	68 (61, 75)	0.150
	“Other treatment” Group	65 (60, 66)	65 (60, 66)	65 (55, 69)	66 (63, 68)	66.5 (55, 72)	0.545
ScVO_2_	Levosimendan Group/change (%) #	58 (38, 74)	68 (31, 81.7)/11.6 (−45, 47) *	73 (56, 89)/24.1 (1, 57) *	76 (55, 89)/37.9 (8, 55) *	79 (45, 89)/39.7 (10, 86) *	0.001
	“Other treatment” Group	65 (50.6, 68)	68.5 (56, 82)/11.2 (2, 21)	70.5 (60, 80)/17.9 (6, 22)	78.5 (63, 83)/22.9 (15, 44)	78.5 (70, 79)/17 (15, 38)	0.056
Lactate	Levosimendan Group/change (%) #	4.5 (2.5, 14.4)	3.6 (1.4, 15.8)/−25.6 (−50, 259) *	2.85 (1.2, 15)/−40.7 (−73, 241)	1.9 (0.9, 15)/−68.3 (−88, −10)	1.45 (0.9, 13)/−68.3 (−88, −10)	<0.0001
	“Other treatment” Group	2.9 (1, 18.9)	4.3 (1, 19.3)/20 (−31, 100)	2.8 (1, 15)/13 (−85, 71)	2.2 (1, 11)/−25 (−89, 169)	1.75 (1.4, 10)/−22 (−93, 43)	0.515
LVEF	Levosimendan Group/change (%) #	15 (10, 20)		20 (10,30)/41,5 (−50, 100) *	27.5 (10, 40)/87.5 (−50, 300) *	50 (30, 68) */225 (−25, 450) *	<0.0001
	“Other treatment” Group/change (%) #	25 (5, 30)		22,5 (5, 30)/0 (−25, 17)	20 (15, 35)/0 (−33, 40)	25 (15, 50)/0 (−33, 100)	0.548
Noradrenaline dose	Levosimendan Group/change (%) #	0.91 (0.49, 1.63)	0.85 (0.45, 1.7)/−9 (−23, 15)	0.68 (0.26, 3.8)/−24 (−78, 280)	0.57 (0, 1.8)/−51.9 (−100, 80)	0.0 (0, 1.2)/−100 (−100, 33) *	0.02
	“Other treatment” Group	0.75 (0.35, 1.67)	0.78 (0.35, 1.6)/3 (−12, 20)	1.15 (0.07, 1.9)/44,4 (−80, 162)	0.89 (0.02, 1.3)/30 (−97, 44)	0.22 (0, 0.5)	0.035

BPM, Beats Per Minute; CVP, Central Venous Pressure; LVEF, Left Ventricular Ejection Fraction; MAP, Mean Arterial Pressure; T_scm,_ time upon the worst EF value during the SCM episode (severe SCM onset); T_12h_, 12 h after severe SCM episode; T_24h,_ 24 h after severe SCM episode; T_72h_, 72 h after severe SCM episode; T_D7,_ Day 7th post severe SCM onset; Pa-vCO_2,_ arteriovenous difference of partial dioxide pressure; SCM, Septic Cardiomyopathy; ScvO_2,_ Superior Vena Cava Oxygen Saturation; TNI, Troponin; the * *p*-value corresponds to the results of the ANOVA; # the changes refer to the percentage change of the value compared to the measurement on T_scm_ upon severe SCM onset.

**Table 3:** Patient outcomes.

	Whole Group (n = 23)	Levosimendan Group (n = 14)	Other Treatment Group (n = 9)	*p*-Value
SCM improvement or resolution [n (%)]	11 (47.8%)	9 (64.3%)	2 (22.2%)	0.054
D_7_ survival [n (%)]	13 (56.5%)	9 (64.3%)	4 (44.4%)	0.424
ICU LOS (days)	11 (1, 48)	14 (1, 47)	7 (1, 48)	0.441
ICU Survival [n (%)]	9 (39.1%)	7 (50%)	2 (22.2%)	0.172

ICU, Intensive Care Unit; LOS, Length of Stay; SCM, Septic Cardiomyopathy.

**Table 4:** A Cox regression analysis model illustrating the effect of several clinical characteristics of septic cardiomyopathy on 28-day mortality.

	HR	95% CI for HR		*p*-Value
		Lower Limit	Upper Limit	
Model overall				0.002
APACHE II score	0.906	0.778	1.055	0.203
SOFA score	1.235	0.994	1.534	0.057
No-levosimendan group	3.756	0.487	28.976	0.204
LVEF on SCM onset	0.785	0.617	1.000	0.050
7-day change in LVEF	0.975	0.958	0.993	0.006

Abbreviations: APACHE II, Acute Physiology and Chronic Health Evaluation; LVEF, Left Ventricular Ejection Fraction; SCM, Septic Cardiomyopathy; SOFA, Sequential Organ Function Assessment.

## Data Availability

Data will be available upon reasonable request.
